# Supplementary material for: The burden of zoonoses in Paraguay: A systematic review
Source: PLoS Negl Trop Dis. 2021 Nov 2;15(11):e0009909. doi: 10.1371/journal.pntd.0009909 (PMC8589157; doi:10.1371/journal.pntd.0009909)
Supplement: S1 Alternative Language Abstract — (DOCX) [file pntd.0009909.s002.docx]

**S1 Alternative Language Abstract - Translation of the Abstract into Spanish by Liz P. Noguera Z.**

**Introducción**

Las zoonosis en países de bajos y medianos ingresos son mayormente subestimadas debido principalmente a las grandes desigualdades que dichos países sufren, generando graves consecuencias en la atención sanitaria. Esto dificulta dimensionar y reducir el impacto de estas enfermedades. Nuestro estudio se centra en Paraguay, donde la industria ganadera es uno de los principales componentes de la economía del país. Este estudio se base en estimar el impacto de enfermedades zoonóticas tanto en humanos como en animales, con el fin de determinar la carga total de dichas enfermedades en la sociedad.

**Metodología/Hallazgos principales**

Se ha realizado una revisión sistemática (incluyendo meta-análisis) con el fin de evaluar la carga zoonótica en Paraguay, considerando informes oficiales y literatura gris sobre la incidencia y prevalencia de enfermedades zoonóticas. En este trabajo, se ha estimado los “*años de vida ajustados por discapacidad*” (AVAD o DALY) y los *"años de vida ajustados por discapacidad zoonótica*” (AVADiz o zDALY) con el objetivo de identificar la diferencia entre el estado de salud actual y la situación de salud deseada de los animales y la población paraguaya en base a 50 enfermedades zoonóticas sugeridas por la OMS (Mundo Organización de la Salud), OIE (Organización Mundial de Sanidad Animal), el Ministerio de Salud y el Servicio Nacional de Calidad y Salud Animal (SENACSA) en Paraguay. De acuerdo con los hallazgos en este estudio, los DALYs totales representan 19,384 (IC del 95%: de 15,805 a 29,733) y los zDALYs 62,178 (IC del 95%: de 48,696 a 77,188). Los agentes patógenos prioritarios en DALYs son: *E. coli, Trypanosoma cruzi, Leishmania spp,* y *Toxoplasma gondi.* Cuando se incluye la carga adicional de enfermedades animales, obtenemos como patógenos más importantes *Brucella spp, E. coli, Trypanosoma cruzi* y *Fasciola hepatica* en los zDALYs.

**Conclusión/Significancia**

Este es el primer estudio en integrar DALYs y zDALYs con importantes hallazgos relacionados al estado de salud en Paraguay. A través de los DALYs y zDALYs, nuestra perspectiva es más integral ya que se considera no solo la salud humana sino también la salud animal; lo cual es importante para establecer prioridades en el control de enfermedades zoonóticas, especialmente en un país donde la producción ganadera contribuye de manera significativa a la economía y al bienestar de la población.
