# Supplementary material for: The burden of zoonoses in Paraguay: A systematic review
Source: PLoS Negl Trop Dis. 2021 Nov 2;15(11):e0009909. doi: 10.1371/journal.pntd.0009909 (PMC8589157; doi:10.1371/journal.pntd.0009909)
Supplement: S4 Table — (DOCX) [file pntd.0009909.s006.docx]

# S4 Table. Auxiliary information of zoonoses used to estimate the YLL, YLD and DALYs.

| **Disease** | **Details** | **Disability weight (95% CI)** | **Average duration** | **References** |
| --- | --- | --- | --- | --- |
| **Brucellosis** | Chronic brucellosis | 0.150 | 75 days | [1–3] |
| **Campylobacteriosis** |  | 0.247 (0.164-0.348) - Severe diarrheal diseases  0.188 (0.125-0.264) - Moderate diarrheal diseases | 10.5 days | [4–6] |
| **Chagas** | Acute Chagas disease | 0.051 (0.032-0.074) | 2 months | [4, 7] |
|  | Treated heart failure due to Chagas disease | 0.049(0.031-0.072) | 20 years |  |
|  | Moderate chronic digestive disease due to Chagas disease | 0.114 (0.078-0.159) |  |  |
| **Cystic echinococcosis** |  | 0.123 | 10 days | [8, 9] |
| **Ehrlichiosis** |  | 0·051 (0·032–0·074) - Acute episode, moderate infectious disease | 28 days | [10–12] |
| **Colibacillosis** |  | 0.247 (0.164-0.348) - Severe diarrheal diseases  0.188 (0.125-0.264) - Moderate diarrheal diseases | 15.5 days | [4, 13] |
| **Fungal skin disease** | *Microsporum canis* | 0.006 (0.002-0.012) - Other fungal skin diseases | 2 - 4 weeks | [4, 14] |
| **Giardiasis** | *Giardia lamblia/G. duodenalis/G. intestinalis* | 0.188 (0.125-0.264) - Moderate diarrheal diseases | 21 days | [15–17] |
| **Hantavirus** | Pulmonary syndrome (HPS) | 0.210 (0.139 - 0.298) | 49 days | [18–22] |
| **Leishmaniasis** | Cutaneous, mucosal and visceral | Cutaneous and mucocutaneous: 0.067 (0.044-0.096)  Visceral (moderate): 0.051 (0.032-0.074) | Cutaneous: 380.5 days  Mucocutaneous: 90 days  Visceral: 198 days | [4, 23–25] |
| **Leptospirosis** |  | 0.0562 (fatal) | 17 days | [26–28] |
| **Myiasis** |  | 0.011  (0.005-0.021)  Symptomatic other skin and subcutaneous diseases | 1 week (without sequelae) | (89,114) |
| **Rabies -post exposure prophylaxis** |  | 0.108 | 60 days | [31] |
| **Salmonellosis** | Non-typhoidal (serovars: Typhimurium and Enteritidis) | 0.133 (0.088- 0.190) | 14 days | [32–34] |
| **Scabies** |  | 0.027 (0·015 – 0·042) | 6 weeks to 1 year | [35] |
| **Toxocariasis** |  | 0.011 (0.005-0.021) - Mild abdominopelvic problems due to hookworm disease | 14.5 weeks | [4, 36] |
| **Toxoplasmosis** | Congenital Toxoplasmosis (CT) | 0.033 -  Chorioretinitis | 10 years | [22, 37, 38] |
| **Tuberculosis** |  | 0.271(0.264 - 0.294) | 7.75 months | [39, 40] |

**References**

1. Rovid Spickler A (2003) Brucellosis.

2. Bosilkovski M, Krteva L, Dimzova M, Vidinic I, Sopova Z, Spasovska K (2010) Human brucellosis in Macedonia-10 years of clinical experience in endemic region. Croat Med J 51:327–336

3. Dean AS, Crump L, Greter H, Hattendorf J, Schelling E, Zinsstag J (2012) Clinical Manifestations of Human Brucellosis: A Systematic Review and Meta-Analysis. PLoS Negl Trop Dis. https://doi.org/10.1371/journal.pntd.0001929

4. Global Burden of Disease Study 2017 (GBD 2017) Disability Weights | GHDx. http://ghdx.healthdata.org/record/ihme-data/gbd-2017-disability-weights. Accessed 30 Jun 2020

5. (2012) The Global View of Campylobacteriosis. Utrecht, Netherlands

6. Acheson D, Allos BM (2001) Campylobacter jejuni Infections: Update on Emerging Issues and Trends. Clin Infect Dis 32:1201–1206

7. Chagas disease (also known as American trypanosomiasis). https://www.who.int/news-room/fact-sheets/detail/chagas-disease-(american-trypanosomiasis). Accessed 14 Aug 2020

8. Torgerson PR, de Silva NR, Fèvre EM, Kasuga F, Rokni MB, Zhou XN, Sripa B, Gargouri N, Willingham AL, Stein C (2014) The global burden of foodborne parasitic diseases: An update. Trends Parasitol 30:20–26

9. Khachatryan AS (2017) Analysis of lethality in echinococcal disease. Korean J Parasitol 55:549–553

10. Ehrlichiosis home | Ehrlichiosis | CDC. https://www.cdc.gov/ehrlichiosis/index.html. Accessed 30 Jun 2020

11. Snowden J, Simonsen KA (2020) Ehrlichiosis. StatPearls Publishing

12. Salomon JA, Haagsma JA, Davis A, et al (2015) Disability weights for the Global Burden of Disease 2013 study. Lancet Glob Heal 3:e712–e723

13. E. coli. https://www.who.int/news-room/fact-sheets/detail/e-coli. Accessed 30 Jun 2020

14. Karimkhani C, Dellavalle RP, Coffeng LE, et al (2017) Global skin disease morbidity and mortality an update from the global burden of disease study 2013. JAMA Dermatology 153:406–412

15. Bartelt LA, Sartor RB (2015) Advances in understanding Giardia: Determinants and mechanisms of chronic sequelae. F1000Prime Rep. https://doi.org/10.12703/P7-62

16. CDC - DPDx - Giardiasis. https://www.cdc.gov/dpdx/giardiasis/index.html. Accessed 6 Jul 2020

17. GIARDIASIS. http://publichealth.lacounty.gov/acd/pubs/reports/annual/cd00/giardia00.pdf. Accessed 10 Jul 2020

18. CDC - Hantavirus. https://www.cdc.gov/hantavirus/. Accessed 30 Jun 2020

19. PAHO/WHO | Hantavirus. https://www.paho.org/hq/index.php?option=com_content&view=article&id=14911:hantavirus&Itemid=40721&lang=en. Accessed 30 Jun 2020

20. Manigold T, Vial P (2014) Human hantavirus infections: epidemiology, clinical features, pathogenesis and immunology.

21. Vial PA, Valdivieso F, Mertz G, Castillo C, Belmar E, Delgado I, Tapia M, Ferrés M (2006) Incubation period of hantavirus cardiopulmonary syndrome. Emerg Infect Dis 12:1271–1273

22. Salomon JA, Vos T, Hogan DR, et al (2012) Common values in assessing health outcomes from disease and injury: Disability weights measurement study for the Global Burden of Disease Study 2010. Lancet 380:2129–2143

23. Leishmaniasis. https://www.who.int/health-topics/leishmaniasis#tab=tab_1. Accessed 30 Jun 2020

24. Piscopo T V, Azzopardi CM (2007) Leishmaniasis (Reprinted from vol 82, pg 649-657, 2006). Postgrad Med J 83:649–657

25. Prevention C-C for DC and (2020) CDC - Leishmaniasis - Resources for Health Professionals.

26. (2017) WHO | Diseases. WHO

27. Torgerson PR, Hagan JE, Costa F, Calcagno J, Kane M, Martinez-Silveira MS, Goris MGA, Stein C, Ko AI, Abela-Ridder B (2015) Global Burden of Leptospirosis: Estimated in Terms of Disability Adjusted Life Years. PLoS Negl Trop Dis 9:e0004122

28. Mga G Global Burden of Leptospirosis: Morbidity, Mortality and DALYs.

29. Francesconi F, Lupi O (2012) Myiasis. Clin Microbiol Rev 25:79–105

30. GBD 2017 Disability Weights (Copy of IHME_GBD_2017_DISABILITY_WEIGHTS_Y2018M11D08-2).

31. Hampson K, Coudeville L, Lembo T, et al (2015) Estimating the Global Burden of Endemic Canine Rabies. PLoS Negl Trop Dis 9:1–20

32. Stanaway JD, Parisi A, Sarkar K, et al (2019) The global burden of non-typhoidal salmonella invasive disease: a systematic analysis for the Global Burden of Disease Study 2017. Lancet Infect Dis 19:1312–1324

33. Salmonella (non-typhoidal). https://www.who.int/news-room/fact-sheets/detail/salmonella-(non-typhoidal). Accessed 30 Jun 2020

34. Saleh S, Van Puyvelde S, Staes A, Timmerman E, Barbé B, Jacobs J, Gevaert K, Deborggraeve S (2019) Salmonella Typhi, Paratyphi A, Enteritidis and Typhimurium core proteomes reveal differentially expressed proteins linked to the cell surface and pathogenicity. PLoS Negl Trop Dis 13:e0007416

35. Karimkhani C, Colombara D V., Drucker AM, Norton SA, Hay R, Engelman D, Steer A, Whitfeld M, Naghavi M, Dellavalle RP (2017) The global burden of scabies: a cross-sectional analysis from the Global Burden of Disease Study 2015. Lancet Infect Dis 17:1247–1254

36. Rovid Spickler A (2004) Toxocariasis.

37. Paul R Torgerson PM WHO | The global burden of congenital toxoplasmosis: a systematic review. https://www.who.int/bulletin/volumes/91/7/12-111732/en/. Accessed 30 Jun 2020

38. Kortbeek LM, Hofhuis A, Nijhuis CDM, Havelaar AH (2009) Congenital toxoplasmosis and DALYs in the Netherlands. Mem Inst Oswaldo Cruz 104:370–373

39. Abdullahi OA, Ngari MM, Sanga D, Katana G, Willetts A (2019) Mortality during treatment for tuberculosis; a review of surveillance data in a rural county in Kenya. PLoS One. https://doi.org/10.1371/journal.pone.0219191

40. Tuberculosis. https://www.who.int/news-room/fact-sheets/detail/tuberculosis. Accessed 30 Jun 2020
