# Supplementary material for: The burden of zoonoses in Paraguay: A systematic review
Source: PLoS Negl Trop Dis. 2021 Nov 2;15(11):e0009909. doi: 10.1371/journal.pntd.0009909 (PMC8589157; doi:10.1371/journal.pntd.0009909)
Supplement: S1 Text — (DOCX) [file pntd.0009909.s007.docx]

# S1 Text. Search terms according to each database.

| **Web of science** |
| --- |
| Paraguay AND (zoonosis OR enfermedad zoonótica OR zoonoses OR zoonotic disease) |
| Paraguay AND (Ehrlichi* OR erliqui* OR leish* OR hantavirus OR Bunyaviridae OR anthrax OR Bacillus anthracis OR rabi* OR Lyssavirus OR brucel* OR toxoplasma OR tuberculosis OR Mycobacterium tuberculosis OR Babesi* OR anaplasma OR Echinococc* OR equinococosis OR hidatidosis OR leptospir* OR rickettsi* OR yellow fever OR fiebre amarilla OR influenza aviar OR gripe aviar OR chagas OR Trypanosoma cruzi OR Streptococcus suis OR streptoco* OR trypanosomosis OR tsetse OR sleeping sickness OR enfermedad del sueño OR filariosis OR Dirofilaria immitis OR tetanos OR Clostridium tetani) |
| Paraguay AND (miasis OR myiasis OR screwworm OR Cochliomyia OR Chrysomya OR Listeria monocytogenes OR lister* OR septicemia hemorragica OR Pasteurella multocida OR Dioctophyma renale OR diocto* OR esparganosis OR S. proliferum OR Campilobacter OR campylobacter OR shiga OR Escherichia coli OR Salmonel* OR VTEC OR STEC OR fasciola OR foodborne trematode infections OR Clonorchis OR clonorchiasis OR Opisthorchis OR opisthorchiasis OR Paragonimus OR paragonimiasis OR Taenia OR cysticercosis OR tapeworm OR roundworm OR trichinell* OR Encefalitis equina venezolana OR Togaviridae OR Alphavirus OR venezuelan equine encephalitis virus OR VEEV OR Burkholderia mallei OR glanders OR muermo OR clamidiosis OR Chlamydophila abortus OR Clamidiosis aviar OR Chlamydophila psittaci OR Scabies OR sarna OR Toxocariasis OR T. canis OR dermatophytes OR sporotrichosis OR Sporothrix OR esporotricosis) |

| **Scopus** |
| --- |
| Paraguay AND ( zoonosis OR zoonoses OR zooonotic AND diseases ) |
| Paraguay AND (leish* OR ehrlichi* OR erliqui* OR anaplasma) |
| Paraguay AND (rickettsi* OR babesi*) |
| Paraguay AND (ántrax OR anthrax OR Bacillus anthracis) |
| Paraguay AND (hantavirus OR Bunyaviridae) |
| Paraguay AND (bovine tuberculosis OR Mycobacterium bovis OR zoonotic tuberculosis) |
| Paraguay AND (brucellosis OR brucelosis OR Brucella abortus) |
| Paraguay AND (toxoplasma OR toxoplasmos*) |
| Paraguay AND (Echinococc* OR equinococosis OR hidatidosis OR hydatidosis) |
| Paraguay AND (Lyssavirus OR rabia OR rabies OR raiva) |
| Paraguay AND (Leptospira OR leptospir*) |
| Paraguay AND (yellow fever OR fiebre amarilla) |
| Paraguay AND (influenza aviar OR gripe aviar OR avian influenza) |
| Paraguay AND (chagas OR Trypanosoma cruzi OR Trypanosomosis OR tripanosomiasis OR tsetse OR sleeping sickness OR enfermedad del sueño) |
| Paraguay AND (Dirofilaria immitis OR filariasis OR filariosis) |
| Paraguay AND (Dioctophym* renale OR dioctofimosis OR dioctophymosis) |
| Paraguay AND (Streptococcus suis OR Streptococcus canis OR Streptococcus equi OR Streptococcus dysgalactiae OR Streptococcus equisimilis OR Streptococcus iniae OR streptococcosis OR estreptococosis) |
| Paraguay AND (Listeria monocytogenes OR listeriosis) |
| Paraguay AND (Cochliomyia OR Chrysomya OR miasis OR myiasis OR screwworm) |
| Paraguay AND (Pasteurella multocida OR septicemia hemorrágica OR Pasteurella aerogenes OR Pasteurella canis OR Pasteurella dagmatis OR Pasteurella pneumotropica OR Pasteurella stomatis) |
| Paraguay AND (S. proliferum OR esparganosis OR sparganosis) |
| Paraguay AND (scabies OR sarna OR Sarcoptes OR dermatophytes OR sporotrichosis OR Sporothrix OR esporotricosis) |
| Paraguay AND (Campilobacter OR campylobacter OR disenteria OR shiga dysentery OR shiga toxin OR Escherichia coli OR VTEC OR STEC OR Salmonella) |
| Paraguay AND ( fasciola OR clonorchis OR clonorchiasis OR opisthorchis OR opisthorchiasis OR paragonimus OR paragonimiasis) |
| Paraguay AND (Taenia OR cysticercosis OR tapeworm OR roundworm OR Trichinella OR trichinellosis OR triquinelosis OR triquinosis OR triquiniasis OR Toxocara OR toxocarosis OR toxocariasis) |
| Paraguay AND (Encefalitis equina venezolana OR Togaviridae OR Alphavirus OR venezuelan equine encephalitis virus OR VEEV) |
| Paraguay AND (clamidiosis OR chlamydiosis OR Chlamydophila abortus OR Clamidiosis aviar OR Chlamydophila psittaci) |
| Paraguay AND (Burkholderia pseudomallei OR Burkholderia mallei OR human glanders OR muermo OR melioidosis) |
| Paraguay AND (Encefalopatía espongiforme bovina OR EEB OR Bovine Spongiform Encephalopathy OR BSE OR Mad Cow Disease OR Creutzfeldt-Jacok disease OR CJD) |
| Paraguay AND (Schistosoma japonicum OR schistosomosis OR schistosomiasis OR esquistosomiasis) |
| Paraguay AND (Tunga penetrans OR Tungiasis OR pique) |
| Paraguay AND (Sarcocystis OR sarcocystosis OR sarcocistosis) |
| Paraguay AND (Fiebre del Valle del Rift OR Q fever OR tularemia OR West Nile fever OR encefalomielitis por Virus Nipah) |
| Paraguay AND (Capillaria hepatica OR capillariasis OR capilariasis) |
| Paraguay AND (Cryptococcus gatti OR Cryptococcus neoformans OR cryptococcosis OR criptococosis OR Cryptosporidium OR cryptosporidiosis OR criptosporidiosis) |
| Paraguay AND (Histoplasma capsulatum OR histoplasmosis) |

| **Ebscohost** |
| --- |
| Paraguay AND ( zoonosis OR zoonoses OR zooonotic AND diseases ) |
| Paraguay AND (leish* OR ehrlichi* OR erliqui* OR anaplasma) |
| Paraguay AND (rickettsi* OR babesi*) |
| Paraguay AND (ántrax OR anthrax OR Bacillus anthracis) |
| Paraguay AND (hantavirus OR Bunyaviridae) |
| Paraguay AND (bovine tuberculosis OR Mycobacterium bovis OR zoonotic tuberculosis) |
| Paraguay AND (brucellosis OR brucelosis OR Brucella abortus) |
| Paraguay AND (toxoplasma OR toxoplasmos*) |
| Paraguay AND (Echinococc* OR equinococosis OR hidatidosis OR hydatidosis) |
| Paraguay AND (Lyssavirus OR rabia OR rabies OR raiva) |
| Paraguay AND (Leptospira OR leptospir*) |
| Paraguay AND (yellow fever OR fiebre amarilla) |
| Paraguay AND (influenza aviar OR gripe aviar OR avian influenza) |
| Paraguay AND (chagas OR Trypanosoma cruzi OR Trypanosomosis OR tripanosomiasis OR tsetse OR sleeping sickness OR enfermedad del sueño) |
| Paraguay AND (Clostridium tetani OR tétanos OR tetanus) |
| Paraguay AND (Dirofilaria immitis OR filariasis OR filariosis) |
| Paraguay AND (Dioctophyma renale OR dioctofimosis OR dioctophymosis) |
| Paraguay AND (Streptococcus OR streptococcosis OR estreptococosis) |
| Paraguay AND (Listeria monocytogenes OR listeriosis) |
| Paraguay AND (Cochliomyia OR Chrysomya OR miasis OR myiasis OR screwworm) |
| Paraguay AND (Pasteurella OR septicemia hemorrágica) |
| Paraguay AND (S. proliferum OR esparganosis OR sparganosis) |
| Paraguay AND (scabies OR sarna OR Sarcoptes OR dermatophytes OR sporotrichosis OR Sporothrix OR esporotricosis) |
| Paraguay AND (Campilobacter OR campylobacter OR disenteria OR shiga dysentery OR shiga toxin OR Escherichia coli OR VTEC OR STEC OR Salmonella) |
| Paraguay AND ( fasciola OR clonorchis OR clonorchiasis OR opisthorchis OR opisthorchiasis OR paragonimus OR paragonimiasis) |
| Paraguay AND (Taenia OR cysticercosis OR tapeworm OR roundworm OR Trichinella OR trichinellosis OR triquinelosis OR triquinosis OR triquiniasis OR Toxocara OR toxocarosis OR toxocariasis) |
| Paraguay AND (Encefalitis equina venezolana OR Togaviridae OR Alphavirus OR venezuelan equine encephalitis virus OR VEEV) |
| Paraguay AND (clamidiosis OR chlamydiosis OR Chlamydophila abortus OR Clamidiosis aviar OR Chlamydophila psittaci) |
| Paraguay AND (Ascaris suum OR Diphyllobothrium) |
| Paraguay AND (Burkholderia pseudomallei OR Burkholderia mallei OR human glanders OR muermo OR melioidosis) |
| Paraguay AND (Encefalopatía espongiforme bovina OR EEB OR Bovine Spongiform Encephalopathy OR BSE OR Mad Cow Disease OR Creutzfeldt-Jacok disease OR CJD) |
| Paraguay AND (Schistosoma japonicum OR schistosomosis OR schistosomiasis OR esquistosomiasis) |
| Paraguay AND (Tunga penetrans OR Tungiasis OR pique) |
|  |
| Paraguay AND (Sarcocystis OR sarcocystosis OR sarcocistosis) |
| Paraguay AND (Fiebre del Valle del Rift OR Q fever OR tularemia OR West Nile fever OR encefalomielitis por Virus Nipah) |
| Paraguay AND (Capillaria hepatica OR capillariasis OR capilariasis) |
| Paraguay AND (Cryptococcus gatti OR Cryptococcus neoformans OR cryptococcosis OR criptococosis OR Cryptosporidium OR cryptosporidiosis OR criptosporidiosis) |
| Paraguay AND (Histoplasma capsulatum OR histoplasmosis) |

| **Pubmed** |
| --- |
| Paraguay AND (zoonosis OR enfermedad zoonótica OR zoonoses OR zoonotic disease) |
| Paraguay AND (Ehrlichi* OR erliqui* OR leish* OR hantavirus OR Bunyaviridae OR anthrax OR Bacillus anthracis OR rabi* OR Lyssavirus OR brucel* OR toxoplasma OR tuberculosis OR Mycobacterium tuberculosis OR Babesi* OR anaplasma OR Echinococc* OR equinococosis OR hidatidosis OR leptospir* OR rickettsi* OR yellow fever OR fiebre amarilla OR influenza aviar OR gripe aviar OR chagas OR Trypanosoma cruzi OR Streptococcus suis OR streptoco* OR trypanosomosis OR tsetse OR sleeping sickness OR enfermedad del sueño OR filariosis OR Dirofilaria immitis OR tetanos OR Clostridium tetani) |
| Paraguay AND (miasis OR myiasis OR screwworm OR Cochliomyia OR Chrysomya OR Listeria monocytogenes OR lister* OR septicemia hemorragica OR Pasteurella multocida OR Dioctophyma renale OR diocto* OR esparganosis OR S. proliferum OR Campilobacter OR campylobacter OR disenteria OR shiga dysentery OR shiga toxin OR Escherichia coli OR VTEC OR STEC OR Salmonella OR fasciola OR foodborne trematode infections OR Clonorchis OR clonorchiasis OR Opisthorchis OR opisthorchiasis OR Paragonimus OR paragonimiasis OR Taenia OR cysticercosis OR tapeworm OR roundworm OR trichinell* OR Encefalitis equina venezolana OR Togaviridae OR Alphavirus OR venezuelan equine encephalitis virus OR VEEV OR Burkholderia mallei OR glanders OR muermo OR clamidiosis OR Chlamydophila abortus OR Clamidiosis aviar OR Chlamydophila psittaci OR Scabies OR sarna OR Toxocariasis OR T. canis OR dermatophytes OR sporotrichosis OR Sporothrix OR esporotricosis) |

| **Google scholar** |
| --- |
| Paraguay AND (zoonosis OR zoonoses OR zoonotic diseases OR enfermedades zoonoticas) |
| Paraguay AND (leish* OR ehrlichi* OR erliqui* OR anaplasma) |
| Paraguay AND (rickettsi* OR babesi*) |
| Paraguay AND (ántrax OR anthrax OR Bacillus anthracis) |
| Paraguay AND (hantavirus OR Bunyaviridae) |
| Paraguay AND (tuberculosis OR Mycobacterium tuberculosis) |
| Paraguay AND (bovine tuberculosis OR Mycobacterium bovis OR zoonotic tuberculosis) |
| Paraguay AND (brucellosis OR brucelosis OR Brucella abortus) |
| Paraguay AND (toxoplasma OR toxoplasmos*) |
| Paraguay AND (Echinococc* OR equinococosis OR hidatidosis OR hydatidosis) |
| Paraguay AND (Lyssavirus OR rabia OR rabies OR raiva) |
| Paraguay AND (Leptospira OR leptospir*) |
| Paraguay AND (influenza aviar OR gripe aviar OR avian influenza) |
| Paraguay AND (chagas OR Trypanosoma cruzi OR Trypanosomosis OR tripanosomiasis OR tsetse OR sleeping sickness OR enfermedad del sueño) |
| Paraguay AND (Dirofilaria immitis OR filariasis OR filariosis) |
| Paraguay AND (Dioctophym* renale OR dioctofimosis OR dioctophymosis) |
| Paraguay AND (Streptococcus suis OR Streptococcus canis OR Streptococcus equi OR Streptococcus dysgalactiae OR Streptococcus equisimilis OR Streptococcus iniae OR streptococcosis OR estreptococosis) |
| Paraguay AND (Listeria monocytogenes OR listeriosis) |
| Paraguay AND (Cochliomyia OR Chrysomya OR miasis OR myiasis OR screwworm) |
| Paraguay AND (Pasteurella multocida OR septicemia hemorrágica OR Pasteurella aerogenes OR Pasteurella canis OR Pasteurella dagmatis OR Pasteurella pneumotropica OR Pasteurella stomatis) |
| Paraguay AND (S. proliferum OR esparganosis OR sparganosis) |
| Paraguay AND (scabies OR sarna OR Sarcoptes OR dermatophytes OR sporotrichosis OR Sporothrix OR esporotricosis) |
| Paraguay AND (Campilobacter OR campylobacter OR disenteria OR shiga dysentery OR shiga toxin OR Escherichia coli OR VTEC OR STEC) |
| Paraguay AND (Fasciola OR fasciolosis OR fascioliasis OR foodborne trematode infections OR Clonorchis OR clonorchiasis OR Opisthorchis OR opisthorchiasis OR Paragonimus OR paragonimiasis) |
| Paraguay AND (Taenia OR cysticercosis OR tapeworm OR roundworm OR Trichinella OR trichinellosis OR triquinelosis OR triquinosis OR triquiniasis OR Toxocara OR toxocarosis OR toxocariasis) |
| Paraguay AND (Encefalitis equina venezolana OR Togaviridae OR Alphavirus OR venezuelan equine encephalitis virus OR VEEV) |
| Paraguay AND (clamidiosis OR chlamydiosis OR Chlamydophila abortus OR Clamidiosis aviar OR Chlamydophila psittaci) |
| Paraguay AND (Burkholderia pseudomallei OR Burkholderia mallei OR human glanders OR muermo OR melioidosis) |
| Paraguay AND (Encefalopatía espongiforme bovina OR EEB OR Bovine Spongiform Encephalopathy OR BSE OR Mad Cow Disease OR Creutzfeldt-Jacok disease OR CJD) |
| Paraguay AND (Schistosoma japonicum OR schistosomosis OR schistosomiasis OR esquistosomiasis) |
| Paraguay AND (Tunga penetrans OR Tungiasis OR pique) |
| Paraguay AND (Sarcocystis OR sarcocystosis OR sarcocistosis) |
| Paraguay AND (Fiebre del Valle del Rift OR Q fever OR tularemia OR encefalomielitis por Virus Nipah) |
| Paraguay AND (Capillaria hepatica OR capillariasis OR capilariasis) |
| Paraguay AND (Cryptococcus gatti OR Cryptococcus neoformans OR cryptococcosis OR criptococosis OR Cryptosporidium OR cryptosporidiosis OR criptosporidiosis) |
| Paraguay AND (Histoplasma capsulatum OR histoplasmosis) |
