# Supplementary figures and images for: The burden of zoonoses in Paraguay: A systematic review
Source: PLoS Negl Trop Dis. 2021 Nov 2;15(11):e0009909. doi: 10.1371/journal.pntd.0009909 (PMC8589157; doi:10.1371/journal.pntd.0009909)

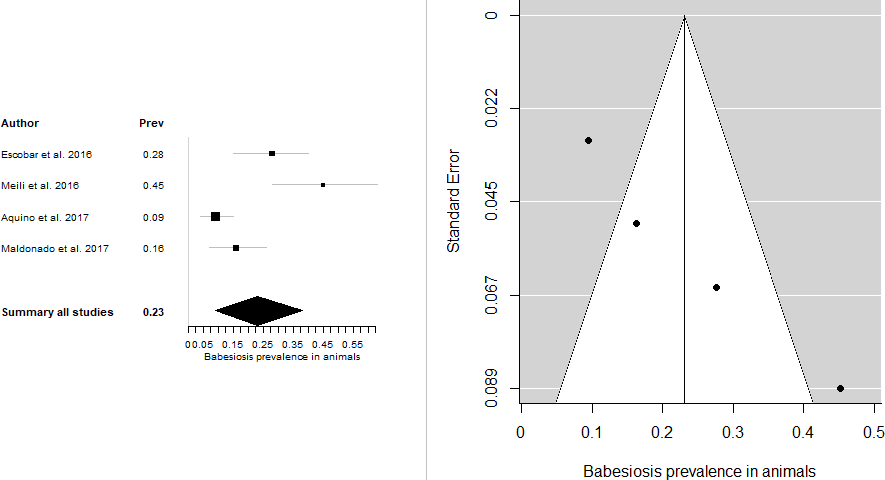

Supplement: S1 File — Fig A: Meta-analysis plots of zoonoses in Paraguay: forest plot and funnel plot of babesiosis in animals. Fig B: Meta-analysis plots of zoonoses in Paraguay: forest plot and funnel plot of ehrlichiosis in animals (dogs). Fig C: Meta-analysis plots of zoonoses in Paraguay: forest plot and funnel plot of leishmaniasis in animals (dogs). Fig D: Meta-analysis plots of zoonoses in Paraguay: forest plot and funnel plot of leishmaniasis in humans. Fig E: Meta-analysis plots of zoonoses in Paraguay: forest plot and funnel plot of leptospirosis in animals. Fig F: Meta-analysis plots of zoonoses in Paraguay: forest plot and funnel plot of leptospirosis in humans. Fig G: Meta-analysis plots of zoonoses in Paraguay: forest plot and funnel plot of rabies in animals. Fig H: Meta-analysis plots of zoonoses in Paraguay: forest plot and funnel plot of scabies in animals. Fig I: Meta-analysis plots of zoonoses in Paraguay: forest plot and funnel plot of toxoplasmosis in humans (ZIP) [file pntd.0009909.s008.zip › S1_File/FigA.tif]

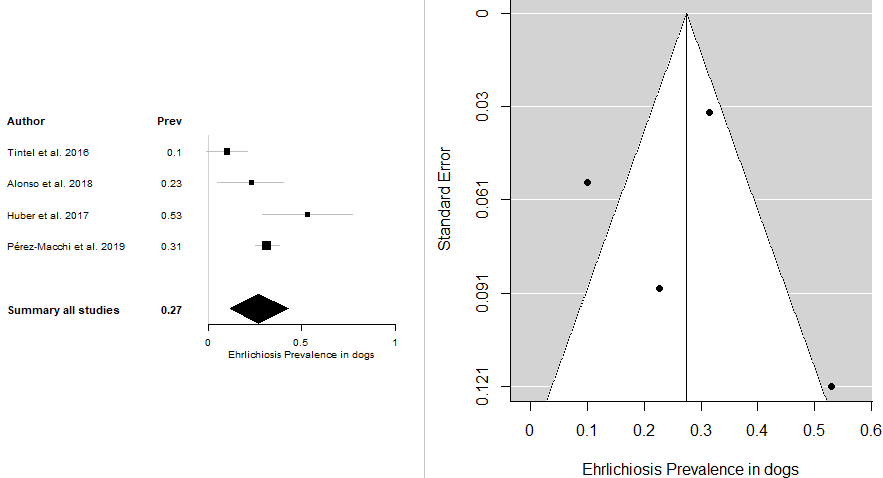

Supplement: S1 File — Fig A: Meta-analysis plots of zoonoses in Paraguay: forest plot and funnel plot of babesiosis in animals. Fig B: Meta-analysis plots of zoonoses in Paraguay: forest plot and funnel plot of ehrlichiosis in animals (dogs). Fig C: Meta-analysis plots of zoonoses in Paraguay: forest plot and funnel plot of leishmaniasis in animals (dogs). Fig D: Meta-analysis plots of zoonoses in Paraguay: forest plot and funnel plot of leishmaniasis in humans. Fig E: Meta-analysis plots of zoonoses in Paraguay: forest plot and funnel plot of leptospirosis in animals. Fig F: Meta-analysis plots of zoonoses in Paraguay: forest plot and funnel plot of leptospirosis in humans. Fig G: Meta-analysis plots of zoonoses in Paraguay: forest plot and funnel plot of rabies in animals. Fig H: Meta-analysis plots of zoonoses in Paraguay: forest plot and funnel plot of scabies in animals. Fig I: Meta-analysis plots of zoonoses in Paraguay: forest plot and funnel plot of toxoplasmosis in humans (ZIP) [file pntd.0009909.s008.zip › S1_File/FigB.tif]

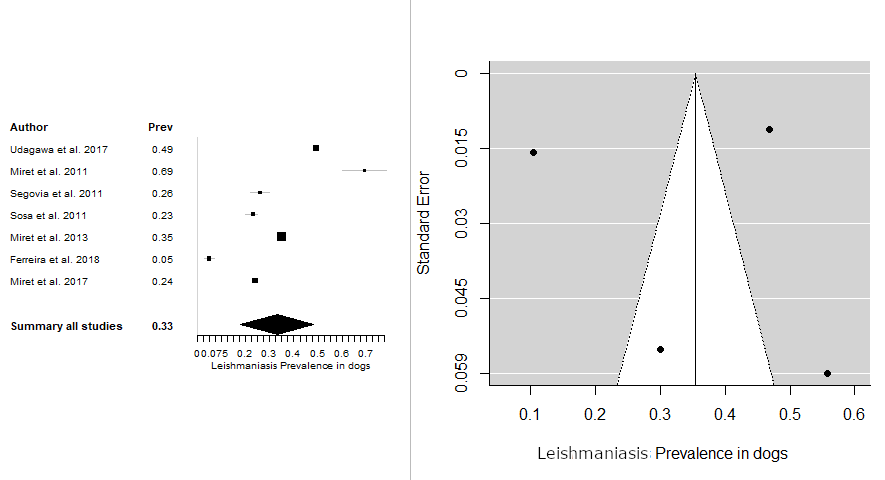

Supplement: S1 File — Fig A: Meta-analysis plots of zoonoses in Paraguay: forest plot and funnel plot of babesiosis in animals. Fig B: Meta-analysis plots of zoonoses in Paraguay: forest plot and funnel plot of ehrlichiosis in animals (dogs). Fig C: Meta-analysis plots of zoonoses in Paraguay: forest plot and funnel plot of leishmaniasis in animals (dogs). Fig D: Meta-analysis plots of zoonoses in Paraguay: forest plot and funnel plot of leishmaniasis in humans. Fig E: Meta-analysis plots of zoonoses in Paraguay: forest plot and funnel plot of leptospirosis in animals. Fig F: Meta-analysis plots of zoonoses in Paraguay: forest plot and funnel plot of leptospirosis in humans. Fig G: Meta-analysis plots of zoonoses in Paraguay: forest plot and funnel plot of rabies in animals. Fig H: Meta-analysis plots of zoonoses in Paraguay: forest plot and funnel plot of scabies in animals. Fig I: Meta-analysis plots of zoonoses in Paraguay: forest plot and funnel plot of toxoplasmosis in humans (ZIP) [file pntd.0009909.s008.zip › S1_File/FigC.tif]

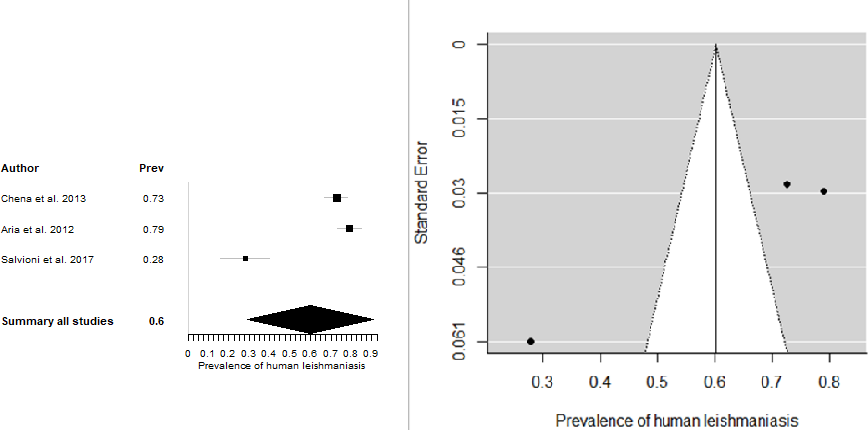

Supplement: S1 File — Fig A: Meta-analysis plots of zoonoses in Paraguay: forest plot and funnel plot of babesiosis in animals. Fig B: Meta-analysis plots of zoonoses in Paraguay: forest plot and funnel plot of ehrlichiosis in animals (dogs). Fig C: Meta-analysis plots of zoonoses in Paraguay: forest plot and funnel plot of leishmaniasis in animals (dogs). Fig D: Meta-analysis plots of zoonoses in Paraguay: forest plot and funnel plot of leishmaniasis in humans. Fig E: Meta-analysis plots of zoonoses in Paraguay: forest plot and funnel plot of leptospirosis in animals. Fig F: Meta-analysis plots of zoonoses in Paraguay: forest plot and funnel plot of leptospirosis in humans. Fig G: Meta-analysis plots of zoonoses in Paraguay: forest plot and funnel plot of rabies in animals. Fig H: Meta-analysis plots of zoonoses in Paraguay: forest plot and funnel plot of scabies in animals. Fig I: Meta-analysis plots of zoonoses in Paraguay: forest plot and funnel plot of toxoplasmosis in humans (ZIP) [file pntd.0009909.s008.zip › S1_File/FigD.tif]

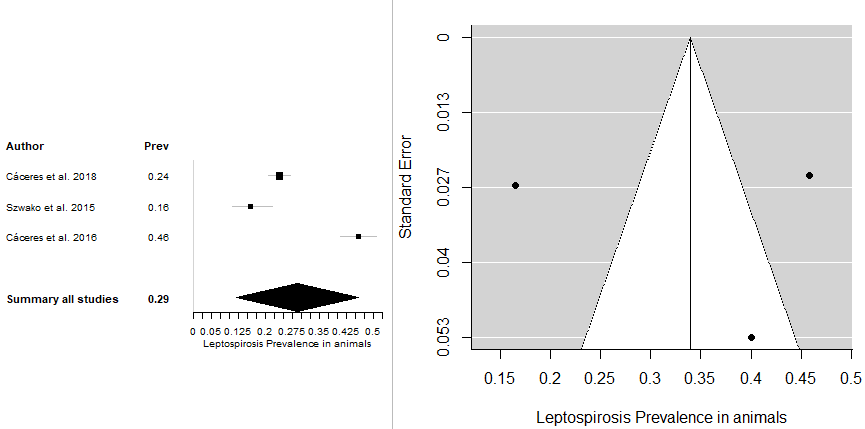

Supplement: S1 File — Fig A: Meta-analysis plots of zoonoses in Paraguay: forest plot and funnel plot of babesiosis in animals. Fig B: Meta-analysis plots of zoonoses in Paraguay: forest plot and funnel plot of ehrlichiosis in animals (dogs). Fig C: Meta-analysis plots of zoonoses in Paraguay: forest plot and funnel plot of leishmaniasis in animals (dogs). Fig D: Meta-analysis plots of zoonoses in Paraguay: forest plot and funnel plot of leishmaniasis in humans. Fig E: Meta-analysis plots of zoonoses in Paraguay: forest plot and funnel plot of leptospirosis in animals. Fig F: Meta-analysis plots of zoonoses in Paraguay: forest plot and funnel plot of leptospirosis in humans. Fig G: Meta-analysis plots of zoonoses in Paraguay: forest plot and funnel plot of rabies in animals. Fig H: Meta-analysis plots of zoonoses in Paraguay: forest plot and funnel plot of scabies in animals. Fig I: Meta-analysis plots of zoonoses in Paraguay: forest plot and funnel plot of toxoplasmosis in humans (ZIP) [file pntd.0009909.s008.zip › S1_File/FigE.tif]

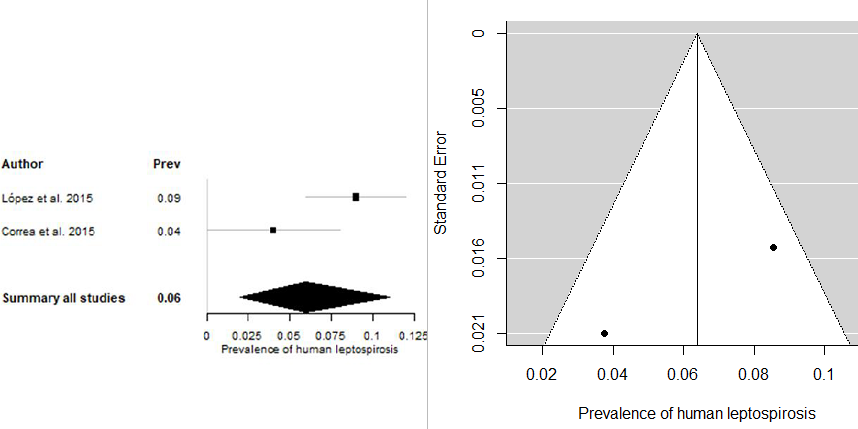

Supplement: S1 File — Fig A: Meta-analysis plots of zoonoses in Paraguay: forest plot and funnel plot of babesiosis in animals. Fig B: Meta-analysis plots of zoonoses in Paraguay: forest plot and funnel plot of ehrlichiosis in animals (dogs). Fig C: Meta-analysis plots of zoonoses in Paraguay: forest plot and funnel plot of leishmaniasis in animals (dogs). Fig D: Meta-analysis plots of zoonoses in Paraguay: forest plot and funnel plot of leishmaniasis in humans. Fig E: Meta-analysis plots of zoonoses in Paraguay: forest plot and funnel plot of leptospirosis in animals. Fig F: Meta-analysis plots of zoonoses in Paraguay: forest plot and funnel plot of leptospirosis in humans. Fig G: Meta-analysis plots of zoonoses in Paraguay: forest plot and funnel plot of rabies in animals. Fig H: Meta-analysis plots of zoonoses in Paraguay: forest plot and funnel plot of scabies in animals. Fig I: Meta-analysis plots of zoonoses in Paraguay: forest plot and funnel plot of toxoplasmosis in humans (ZIP) [file pntd.0009909.s008.zip › S1_File/FigF.tif]

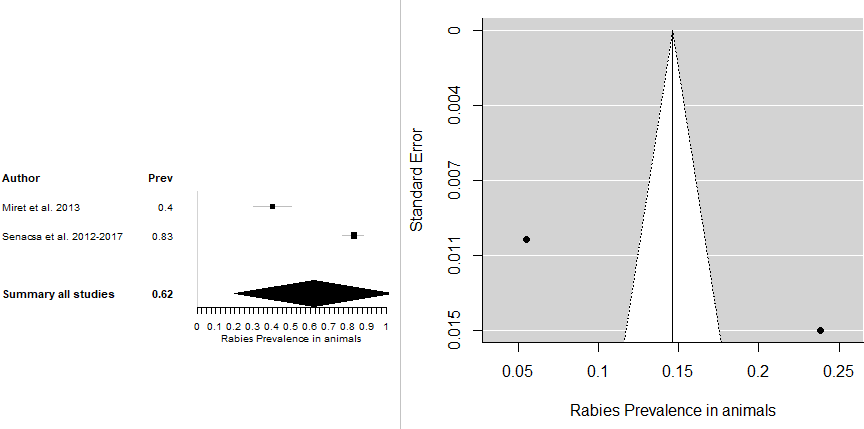

Supplement: S1 File — Fig A: Meta-analysis plots of zoonoses in Paraguay: forest plot and funnel plot of babesiosis in animals. Fig B: Meta-analysis plots of zoonoses in Paraguay: forest plot and funnel plot of ehrlichiosis in animals (dogs). Fig C: Meta-analysis plots of zoonoses in Paraguay: forest plot and funnel plot of leishmaniasis in animals (dogs). Fig D: Meta-analysis plots of zoonoses in Paraguay: forest plot and funnel plot of leishmaniasis in humans. Fig E: Meta-analysis plots of zoonoses in Paraguay: forest plot and funnel plot of leptospirosis in animals. Fig F: Meta-analysis plots of zoonoses in Paraguay: forest plot and funnel plot of leptospirosis in humans. Fig G: Meta-analysis plots of zoonoses in Paraguay: forest plot and funnel plot of rabies in animals. Fig H: Meta-analysis plots of zoonoses in Paraguay: forest plot and funnel plot of scabies in animals. Fig I: Meta-analysis plots of zoonoses in Paraguay: forest plot and funnel plot of toxoplasmosis in humans (ZIP) [file pntd.0009909.s008.zip › S1_File/FigG.tif]

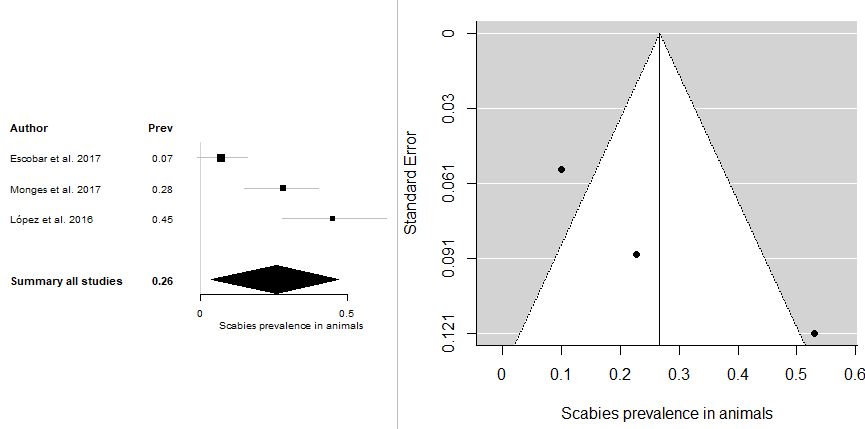

Supplement: S1 File — Fig A: Meta-analysis plots of zoonoses in Paraguay: forest plot and funnel plot of babesiosis in animals. Fig B: Meta-analysis plots of zoonoses in Paraguay: forest plot and funnel plot of ehrlichiosis in animals (dogs). Fig C: Meta-analysis plots of zoonoses in Paraguay: forest plot and funnel plot of leishmaniasis in animals (dogs). Fig D: Meta-analysis plots of zoonoses in Paraguay: forest plot and funnel plot of leishmaniasis in humans. Fig E: Meta-analysis plots of zoonoses in Paraguay: forest plot and funnel plot of leptospirosis in animals. Fig F: Meta-analysis plots of zoonoses in Paraguay: forest plot and funnel plot of leptospirosis in humans. Fig G: Meta-analysis plots of zoonoses in Paraguay: forest plot and funnel plot of rabies in animals. Fig H: Meta-analysis plots of zoonoses in Paraguay: forest plot and funnel plot of scabies in animals. Fig I: Meta-analysis plots of zoonoses in Paraguay: forest plot and funnel plot of toxoplasmosis in humans (ZIP) [file pntd.0009909.s008.zip › S1_File/FigH.tif]

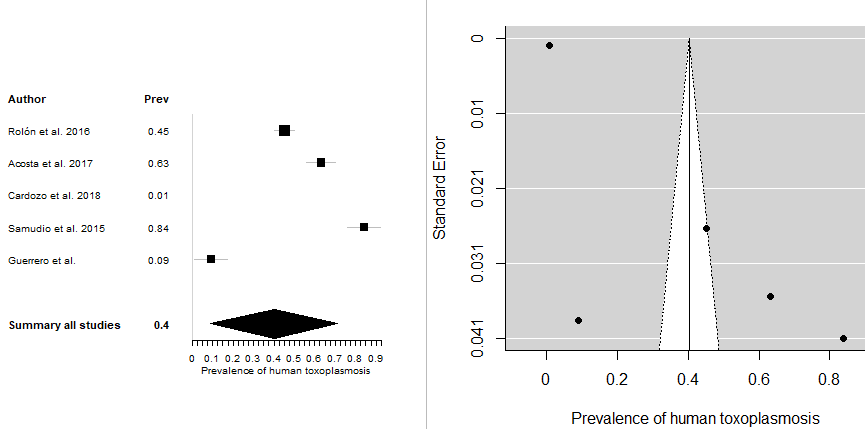

Supplement: S1 File — Fig A: Meta-analysis plots of zoonoses in Paraguay: forest plot and funnel plot of babesiosis in animals. Fig B: Meta-analysis plots of zoonoses in Paraguay: forest plot and funnel plot of ehrlichiosis in animals (dogs). Fig C: Meta-analysis plots of zoonoses in Paraguay: forest plot and funnel plot of leishmaniasis in animals (dogs). Fig D: Meta-analysis plots of zoonoses in Paraguay: forest plot and funnel plot of leishmaniasis in humans. Fig E: Meta-analysis plots of zoonoses in Paraguay: forest plot and funnel plot of leptospirosis in animals. Fig F: Meta-analysis plots of zoonoses in Paraguay: forest plot and funnel plot of leptospirosis in humans. Fig G: Meta-analysis plots of zoonoses in Paraguay: forest plot and funnel plot of rabies in animals. Fig H: Meta-analysis plots of zoonoses in Paraguay: forest plot and funnel plot of scabies in animals. Fig I: Meta-analysis plots of zoonoses in Paraguay: forest plot and funnel plot of toxoplasmosis in humans (ZIP) [file pntd.0009909.s008.zip › S1_File/FigI.tif]
