# Supplementary material for: The burden of zoonoses in Paraguay: A systematic review
Source: PLoS Negl Trop Dis. 2021 Nov 2;15(11):e0009909. doi: 10.1371/journal.pntd.0009909 (PMC8589157; doi:10.1371/journal.pntd.0009909)
Supplement: Attachment — Submitted filename: Response to reviewers BZD Paraguay.docx [file pntd.0009909.s009.docx]

**Manuscript ID:** PNTD-D-20-01638

**Manuscript title:** "The Burden of Zoonoses in Paraguay: A Systematic Review"

Dear Editors,

We thank the reviewers for their time and helpful comments. We have addressed their concerns to improve the manuscript	.

**Methods**

Reviewer #1: The hypothesis is weakly stated as "zoonoses represent a substantial health burden in Paraguay" but to my liking this is not testable. Reference to GDP or other cost impact would have relevance for the costs of zoonoses.

The PRISM approach describes the population of literature surveyed, and demographics for Paraguay are described. The sample size is adequate; statistical testing was not applied nor appropriate.

**Answer:** The burden of disease is usually calculated as DALY or equivalent, so we are not certain why references to GDP (a monetary metric) would be relevant. We have however posed the hypothesis in terms of the total burden or impact of infectious diseases in Paraguay, which we hope will give greater strength and clarity to the hypothesis. The DALYs take part of the perspective from well-being economy, a new vision where money is not an end but a means to an end. Therefore, our aim is not to measure the GDP, but the DALYs.

**Results**

Reviewer #1: The analysis approaches the intended objective of the article (valuation of zoonoses) but there could be more made of the results.

For example, I don’t see how the results from calculating DALYs and ALEs are integrated or at least assessed in unison. In other words, how did these results from ALEs inform our knowledge of DALYs? Surely there is more of a story to tell other than adding to the total financial impact. If the authors feel this was addressed in the article, it needs to be summarized and highlighted in the Discussion towards the end or in the Conclusions.

**Answer:** DALYs and ALEs are integrated as zDALYs. We added the formula to the methodology in case it was not clear. ALEs are the equivalent impact as the DALY on animal owners. This is presently estimated through time tradeoffs (i.e., the time taken to earn the money to replace the animal loss and hence converted to a DALY equivalent). Thus, they are integrated. For instance, the DALYs of brucellosis is 245, but when we also consider the ALEs, brucellosis has 7,291 zDALYs, being the highest value (see Table 1). That is an example of how the results vary if we only consider DALYs. We also included some clarifications in the discussion and conclusion.

Reviewer #1: I find that the discussion on the impact on livelihoods is thin other than to summarize that the diseases are present, recorded, and the authors can confirm that their findings reflect what is in the literature they cite (or in medical records from Paraguay). There is no wider discussion on the social burden of animal diseases in terms of direct or indirect influence on daily decision making that impacts livelihoods for example (e.g., avoidance of some species due disease risk could have economic impact on family income), or impact on family nutrition. Those are just two ideas, but those wider implications of the GBADs seem to be missing in the discussion.

**Answer**: We aimed to show the burden of zoonoses as a DALY equivalent by including the burden due to animal diseases. In GBD, as a comparison, the costs of medical services and the wider social burden (e.g., loss of income, costs of care) are not included in the GBD estimates. So, whilst that might make sense in a future study, it is beyond the scope of the present study. However, we described a bit in the discussion and conclusion.

Reviewer #1: I also wonder why the distributions presented in the results were not discussed in more detail. Are the authors satisfied with those distributions, do they match other findings, is there more particular work to do with regards to simulating distributions, what are the shortcomings of applying those distributions to policy work, etc. There was also no apparent effort to apply sensitivity analysis - a critical feature of robust simulation studies that is missing as far as I can tell.

**Answer:** We were studying how we could apply sensitivity analysis to this study. However, it was not feasible since we did not assess any risk, and we did not make a decision-analysis or a cost-benefit analysis either. Usually, sensitivity analysis is used to see which factors are most responsible for generating high/low values of the output for Monte Carlo analysis, and that was not the aim of our analysis. We did not model the dynamic transmission of diseases, but we estimated DALYs based on the cases that we have found in the systematic review. We did not work on an experimental design either. We slightly described why we used each distribution in the methodology.

For the burden of disease estimates, initially, most sensitivity analyses were undertaken for age-weighting and discount rate values, but since these are no longer used it is not relevant (Mathers, C.D., Salomon, J.A., Ezzati, M., Begg, S., Vander Hoorn, S. and Lopez, A.D., 2006). Sensitivity and uncertainty analyses for the burden of disease and risk factor estimates. Global burden of disease and risk factors. Washington (DC): The International Bank for Reconstruction and Development / The World Bank; New York: Oxford University Press; pp399-426.).

Since we used the data that we found through the systematic review, we have not undertaken a simulation study. Therefore, we cannot perform a sensitivity analysis of a simulation study that we have not done. However, we undertook some sensitivity analysis in terms of the burden of zoonosis, although it was not explicitly stated in the text. Mathers et al. in their methodological paper for the burden of disease estimates for sensitivity analysis, they simply illustrated the relative contribution of each risk factor to the total burden. Hence, it is straightforward to see which risk factors or diseases have the greatest contribution to the burden of disease. Consequently, how the disease burden is sensitive to uncertain data for each disease or risk factor examined. We have added additional text in the manuscript to explain this.

Reviewer #2: Fig 1 map is wrong and not very useful, other countries in the region are missing, labeling is hard to read. A more informative map should be not only more complete, but should incorporate geographical data correlating to the data discussed in the text. Paraguayan regions are mentioned throughout the manuscript and it would be helpful for the reader to easily locate them on this map.

**Answer:** After consideration, we decided to exclude the map since its added value was considered insufficient for this paper.

**Conclusions**

Reviewer #1: See some of my previous comments.

Conclusions are very general and supported by the data but seem cursory relative to what else might be addressed.

Use of these data to advance our understanding falls short of what is promised in L79-82 although this is woven into the discussion of misdiagnosis, under estimation, under ascertainment and (this was not clearly noted but was suggested) access to health care for marginalized and remote communities. Those are all good points that need to be brought to conclusion - can these results influence policy in some way to correct those problems?

**Answer:** We believe that our results show the utility of assessing the burden of human and animal health to society on the same scale. This is the main conclusion. We have rewritten the conclusions in a short succinct form to illustrate this. We hope this is now clear.

**Summary and General Comments**

Reviewer #1: This research has promise to present more robust and extended findings if my comments are taken into consideration. As it stands is presents more as a catalogue of estimations with reflection on which diseases are already noted as zoonotic and a burden in Paraguay. That seems to be the overall weakness of the paper

**Answer:** We added more content to the discussion. We agree that the findings of our study are a subset of a complex system of society, and there are more to do (always are more). However, we think with this study, we provide evidence about how zoonoses vary according to which perspective is studied and treated in a country, having a different impact. We did what we can with the data available being scarce in Paraguay compared to other countries in the region such as Brazil or Argentina.

L57: Reviewer #1: don’t think Fig 1 is particularly helpful considering how much page space this colour illustration occupies. Readers will either know where Paraguay is located, or can find this quickly on the internet. There is nothing to indicate livestock density on this map or relative economic value compared to neighbours, which might have made it more interesting.

**Answer:** Unfortunately, we do not have GPS points associated with livestock distribution in each country of South America to plot it on a map. After consideration, we decided to exclude the map since its added value was considered insufficient for this paper.

L85: Reviewer #1: A few of the zoonoses chosen could be more narrowly defined (E. coli, tuberculosis, and avian influenza for example). You do note that for some diseases this is not available but surely you there is some reference to these distinctions in the literature. For a paper that addresses zoonoses, this seems a critical point of interest. Tuberculosis from M. bovis vs. M. tuberculosis, or HPAI vs. non-pathogenic AI will change weighting factors and output measurements. This should be addressed in general somewhere in your article (cf. my comment L148).

**Answer:** We described those diseases in the discussion.

L98: Reviewer #1 Your PRISMA approach is a well accepted approach and you have conducted what appears to be an extensive search. However, with so many diseases listed compared to the number of articles, I wonder if you might consider a figure that shows clusters of disease over time (e.g., bacterial, viral, common, rare, self-limiting, fatal without intervention, etc.)

**Answer:** We added a new graph that we hope to help to better synthesize this suggestion.

L133, L146: Reviewer #1: Equations would help here. These are not complicated concepts but the explanations are hard to follow¨ for someone not familiar with the equations.

**Answer:** We added more formulas to facilitate the reading.

L148: Reviewer #1: Further to my comment L85, do you not have this distinction for Paraguay? It would be good to know if that is the case. Using all of the Americas as a reference is a pretty wide reference base.

**Answer:** Unfortunately, we do not have this distinction for Paraguay. The closest reference we had was for the Americas.

L170: Reviewer #1: I am not sure from reference where your pet values came from – no mention pet market sources or of social media that I can see in your S2 info. (The livestock sources seem reliable and reasonable.)

**Answer:** In Paraguay, there is not an official source for the prices of pets in the market. Pages such as the “Paraguayan Kennel club” do not have this data. In this country, we usually try to contact dog breeders through social media (mainly Facebook) and sale pages (such as “hendyla”- it is a kind of “Amazon” or “eBay” website but for Paraguay). Since, we did not want to promote any private company or breeder names in a scientific paper, we omitted brands. However, we mentioned it in a general way if some people would like to search by themselves.

L172: Reviewer #1: With respect to simulation, your terminology gets a bit sloppy. Technically, uncertainty cannot be estimated because you do not know either probability or outcome, but you could estimate risk (the product of probability and outcome). What are you estimating? Risk of …? This needs to be stated clearly in the first sentence of the paragraph starting at L172.

Why did you decide on beta, gamma, etc. distributions? Expert opinion, best fit (and then how and of what other distributions), past experience? Why were some diseases modeled under two or more distributions and others only one? A lot of assumptions seem to be missing here. Seed, underlying estimator (MLE, etc.), etc.

**Answer:** We chose models according to the best fit for the data available. We described more in the methodology. For example, estimates of prevalence include a nominator (number affected and denominator (sample size). These are then used to construct a Beta distribution to model the population prevalence from the sample. The beta distribution therefore is a suitable model for the random behavior of percentages and proportions. Likewise, the generalization to multiple categories is a Dirichlet distribution, which was used for the multiple categories of Leishmaniasis. These are examples of why we chose the distributions and have provided further explanation in the text.

L174: Reviewer #1: “For each zoonotic disease, we simulated 10000 draws from the probability distribution of the parameters”- Reviewer #1: I believe what you state as “draws” should be iterations.

**Answer:** yes, thank you! I replaced it. Sampled is also a better term then simulated.

L182: Reviewer #1: Is this in reference to data you refer to previously or is this a new reference? Please clarify and if new, information regarding those sources is needed.

**Answer:** this is in reference to the previous data.

L184: Reviewer #1: Why were these particular diseases selected?

**Answer:** Only for those diseases, we had enough/suitable data to perform a meta-analysis. Ergo each of those diseases had more than two studies, making feasible a meta-analysis.

L185: Reviewer #1: Is the “random effect model” approach something all readers should know? Some clarity is needed here.

### **Answer:** There are two main models in meta-analysis, which are: 1) the fixed-effect model (common-effect model) and 2) the “random effect model.” We thought that it would be useful to mention it. We added one more line in the description of the methodology to clarify.

L195: Reviewer #1: Unless you tested for significance, replace that word with “substantial”. Is there a reason to account for this substantial increase?

**Answer:** Thank you! I corrected the word. We also added to the discussion the plausible reason related to the increase of scientific publications.

L234: Reviewer #1 as well as differentiation between bacterial species and their relative impact on human vs. animal populations. It seems to me this is the Achilles heel of the research – interesting from a global conclusion but weak specificity of local conclusions (to use the modeling reference to global vs. local optima points).

**Answer:** When the zoonotic disease (including bacterial, viral, parasitic, or fungal species) affected only humans, we used DALYs. When the disease also affected animals, we used ALEs which was integrated into the zDALYs. Concerning the modeling reference to global vs. local optima points, we see that this is an extension of the comment about the sensitivity analysis. Please, see our previous answer.

L241: Reviewer #1 Can this not be incorporated into your estimation of the impact of “tuberculosis” somehow, or perhaps model both TB and bTB? I see you have approached that notion in L337-338.

**Answer:** we added more details to the discussion.

L249-252: Reviewer #1 I don’t see the point of this paragraph if these individuals emigrated (out of Paraguay). Please add clarification of relevance.

**Answer:** When people emigrate (especially sick people), this reduces the disease incidence in a country because the diagnosis is conducted elsewhere, and it influences the under-ascertainment of zoonoses in Paraguay. However, we deleted this section due to the emigration rate is approximately 10% compared to the natural rate of population increases and therefore will have a negligible effect on the disease burden.

L373: Reviewer #1 Agreed, but neither is the emotional value of human life incorporated into DALYs. As for wild animals, you could have estimated marketable valuation or value based on economic substitution.

**Answer:** We agree, but that is why ALEs differ from DALYs. You can include more than financial value in this indicator (ALE) to have a holistic time trade-off to replace the animal from the human perspective. Even for pets, it was difficult to find prices in Paraguay. In contrast, there are no economic values for wild animals since it is a felony to market wild animals in this country (but it happens on the black market). The value of wild animals based on economic substitution is complex because we must analyze its ecological value, whether it is a threatened species and so forth. This is interesting for future studies.

L392-400: Reviewer #1 A substantial burden from rabies is the cost to the public sector or PEP treatment as well as dog capture, neuter, vaccinate, and release programs. Lots of articles in the rabies literature to draw on from S. America that you could use just to note that is a cost not incorporated in your study. You might start with: de Carval et al. (2018) Rabies in the Americas: 1998-2014. PNTD. <https://doi.org/10.1371/journal.pntd.0006271>. Chile is now considered free of dog rabies, but it took a strong and costly campaign to get there – should be data on that too.

**Answer:** We agree that the costs of rabies are more than PEP, but the study was in Paraguay, and the government does not spend money on capture or neuter programs, but vaccination which is already included in the PEP. Only responsible owners take care of animal vaccination costs in most cases, but there are no official records. However, we mentioned that there are more costs than PEP to prevent and control rabies. This is also worthy of further studies. We also read the paper that you suggested, and according to the introduction, researchers of that study included Paraguay in it ([<https://doi.org/10.1371/journal.pntd.0006271>](https://doi.org/10.1371/journal.pntd.0006271)). However, when we went deeper to find actual data of Paraguay, we realized that all the data of the countries they keep it anonymously ending up with no identifiable data for Paraguay or any other neighboring countries. Also, we consider that we would introduce more bias if we only account for more costs for certain diseases than others.

Reviewer #2: I would like to discuss some points that were not clear for me: In cases of comorbidity due to more than one infection, how was the employed criteria?

**Answer:** There was not common to find reports of comorbidity of zoonoses but of other infectious diseases such as HIV. In those cases, we only considered zoonoses, excluding other non-zoonotic infectious diseases. When the study consisted of comorbidity of a “case study” of zoonotic diseases (with only one case or less than five cases, we excluded them).

Reviewer #2: I agree that “The five most important pathogens of DALYs in decreasing order constitutes E. coli, Trypanosoma cruzi, Leishmania spp, Toxoplasma gondii and Campylobacter spp. They are responsible for 75% of the disease burden.” However, E. coli and T. cruzi are by far, the most important pathogens, they should be discussed separately since these two pathogens represent a half of the total disease burden.
Should be considered T. cruzi to discuss zDALY? As far as I know, Chagas disease doesn’t affect cattle, poultry, pigs, sheep, horses, goats or any other economically important animal.
More detail is necessary to establish Paraguay as a unique case for this study. When appropriate, available information from other countries should be included for comparison. If it is the purpose of the authors, as stated, to uncover clues in order to set disease control priorities, cross-country comparisons become even more essential. What is the burden of the analyzed zoonoses in neighboring countries? What is the disease burden in non-neighboring countries which share the same type of economy heavily skewed towards agricultural production?

**Answer:** We did consider not only livestock but also pets for our study. For that reason, we included Chagas in our research since dogs also can be affected by this disease. We talked more about *E. coli* and *T. cruzi* in the discussion.

We aimed to focus on Paraguay since it is the least studied country compared to others in the Southern Common Market (MERCOSUR). Focusing only on a country, let us conduct deeper research as we did. Besides, this study was financed by the Paraguayan government, and as a first stage, we consider it appropriate to introduce the burden of zoonotic diseases in this country, including zDALYs. The cross-country comparison is worthy of future study, but with the same methodology to have comparable results.

Reviewer #2: Minor comments:
 Large sections (particularly in the introduction) should be revised for grammar and syntax. Oxford commas are inconsistently used throughout.

Line 11: Reviewer #2: Change “this difficult to gauge”… by “it is difficult to gauge…”

**Answer:** Sorry! I forgot the verb

Line 65: Reviewer #2: losses

**Answer:** Thank you! Changed it!

Line 166: Reviewer #2: Was the economic value of cattle estimated in export prices or prices for the local market, or a combination of both?

**Answer:** It was calculated based on the local market.

Line 167: Reviewer #2: “In respect of pets” change by “with respect to pets”

**Answer:** both expressions are well-accepted in English.

L262. Reviewer #2: It would be useful to mention the legal status of disclosure of risks to workers in the country in this context.

**Answer:** There is a law relatively new (from 2017) with rules and procedures set to protect workers from different risks when they perform their functions. However, it is only the first stage presented in a general way. (More details, https://www.bacn.gov.py/leyes-paraguayas/9127/ley-n-5804-establece-el-sistema-nacional-de-prevencion-de-riesgos-laborales).

Line 285: lose

**Answer:** Thank you! Corrected

Line 383: Discussion of animal bites seems out of place outside of the context of rabies.

**Answer:** We can delete that part, but we considered that animal bites are an introduction to rabies, they are in the same paragraph.

We apology for all the grammatical errors. We amended all the errors that we have found.
