# Supplementary material for: The burden of zoonoses in Paraguay: A systematic review
Source: PLoS Negl Trop Dis. 2021 Nov 2;15(11):e0009909. doi: 10.1371/journal.pntd.0009909 (PMC8589157; doi:10.1371/journal.pntd.0009909)
Supplement: Attachment — Submitted filename: Response to reviewers BDZ in Paraguay.docx [file pntd.0009909.s010.docx]

**Manuscript ID:** PNTD-D-20-01638

**Manuscript title:** "The Burden of Zoonoses in Paraguay: A Systematic Review"

Dear Editors,

We thank again the reviewers for their time and helpful comments. We have addressed their concerns to improve the manuscript

| **Methods** | |
| --- | --- |
| **Reviewer’s comments** | **Answers** |
| Reviewer #1: I am finding it difficult to locate the changes, but I think I succeeded. The version I was sent has no changes indicated in any way. Nor do the authors indicate on what line(s) changes were incorporated in the new manuscript. This would have been helpful. I will refer to my previous comments and author replies.  The authors' response focusing on GDP misses my earlier point - I did not suggest you measure GDP (which, by the way, is an economic metric, not monetary metric). I suggest you need to incorporate some sort of standardized cost measure which would be consistent with your frequent reference to the economic value and market valuation of animals and their use. This to me is one of the weaknesses in general of adopting DALYs or modified DALYs - animals are property with market value but people are not.  This boils down to a philosophical and intellectual debate regarding how to measure and incorporate market valuation. DALYs and your zDALY certainly help, but the wider social impact of the market losses is not captured (e.g., food security and all the other SDGs). I suggest you simply add a line that notes that somewhere in your paper (end of Discussion or Conclusions perhaps). No one has achieved that in a single index and that is your challenge. | We are sorry! We sent to the journal two versions of the manuscript, one of them with track changes. Since we thought the reviewers also have access to the document with track changes, we did not specify which lines we modified in our answers (because we consider it redundant). However, we take your suggestion for this time and next opportunities.  Regarding your comment, animals that have a market value have been converted to a time trade-off. We used their monetary value to estimate the time needed to earn the money that the animal is worth. The same is applied in YLDs for DALYs. YLDs are the time trade-off equivalent for non-fatal diseases.  Anything that adversely affects humans but is not fatal (disease or injury morbidity, loss of animal) is essentially valued in terms of time trade-off for years of life.  We added your suggestion to the conclusion L627-629. |
| Reviewer #2: objectives are clear, and the study design is in agreement with the stated objectives. The population is adequate to test the hypothesis. In the new version submitted, the methodology employed is better explained which makes it clearer. | Thank you! |
| **Results** | |
| **Reviewer’s comments** | **Answers** |
| Reviewer #1: Again, it's quite difficult to find the specific changes without either TrackChanges or author reference to the line(s) where changes were made. In future, I suggest the lead author indicate to reviewers what lines in the new manuscript incorporate changes.  I cannot see where you discuss the social burden of disease in your discussion or conclusion. It may well be beyond the scope of your study, but I do think it bears mentioning as a weakness of current methods of estimation of GBADs. If not already incorporated, please add to the Discussion in a clearly stated sentence. There are many such methods used in development/ growth/ welfare economics, but just mentioning that weakness (absence) I think is important. | We already answered above.  We tried to cover along with the discussion some social issues related to the SDGs such as health, welfare, poverty, education, illiteracy, hunger, malnutrition, inequality, access to work, among others. Also, we mentioned the weakness of this method. |
| Reviewer #2: to clarify, new graph and figure were added to show clusters of disease (publications) from 2000 to 2020 and the relative impact of diseases in the country (DALY, ALE and zDALY) | Fig. 3 was the new graph that we added, where we show how the percentage of each disease varies according to the approach. For example, in Fig3A, we see that the priorities are colibacillosis (27.1%), Chagas (26.9%), Leishmaniasis (10.6%) for humans through the DALYs. On the other hand, in Fig 3B, the priorities change based on the animal disease burden through the ALEs; so, we have Brucellosis (43.1%), Ehrlichiosis (21.9%), and Fascioliasis (21.9%). In Fig 3C, when we combine DALYs + ALEs = zDALYs, we have as priorities Brucellosis (20%), Colibacillosis (17.4%), Chagas (14.8%). |
| **Conclusions** | |
| Reviewer #1: I think your Conclusions is better stated in your revised manuscript. | Thank you both! |
| Reviewer #2: Conclusions were rewritten and looks more clear. They are supported by presented data |  |
| **Summary and General Comments** | |
| Reviewer #1: Other changes to the manuscript (e.g., added graph, equations, etc.) have improved the manuscript. |  |
| **Reviewer’s comments** | **Answers** |
| Reviewer #2: In this paper, the authors quantify the impact of different pathogens in human and animal disease burden in Paraguay, through the estimation of DALY and DALYz parameters respectively. The methodology is now better explained and clearer in this new version of the manuscript. The Discussion Section has been also improved. The authors well discuss the limitations of the methods and the different possible causes of underestimation or overestimation of the presented data. Moreover, each one of the pathogens causing the studied zoonoses are now described in the discussion. In this context, I think that the discussion is now quite long (too much). The authors should focus on the description of the most relevant zoonoses (the high priority) in the study, those with higher DALY and DALYz values. The study of the burden of zoonosis diseases and the interconnection between people and animals will help to develop optimal health outcomes, prioritizing those illness with higher impact in human and animal health minimizing economic losses. For that reason I think that this work is very interesting to be published but I strongly suggest shortening the section Discussion to accept the manuscript.  minor comments:  E coli must be defined by Escherichia coli by the first time Line 234. From results in Table 1, one the most relevant pathogens, considering the zDALY calculation was T. cruzi, however, there is no ALE data in the table (from dogs) so in this case, zDALY is the same as DALY. Is zDALY discussion relevant in this case? In figure 3a, 3b and 3c, What is the meaning of the numbers in the right upper corner? are they percentages of the estimated total DALY, Ale and zDALY respectively? Please, explain | When we have the absence of ALE estimations for a specific zoonotic disease (e.g., *T. cruzi*), it means there is a lack of study of that disease in animals. As “absence of evidence is not evidence of absence,” we consider important to discuss other possible sources of disease infections. The idea is not to study zoonoses as isolated cases only in humans or animals but through a holistic approach.  We deleted some parts of the discussion (deleted lines: 354, 377, 378, 399-402, 473-479, 597, 598)  L234: Corrected  Since Chagas has a high burden on the human population is also important to suggest studies in animals, especially when there is no data. We found Chagas in pets in neighboring countries. So, the question is, why not in Paraguay? Pets might also be a source of Chagas, not just the triatome, but more studies are needed to answer that. That is why we believe it is important to mention it as well.  We already explained a bit the Fig. 3a, 3b, and 3c above. The numbers in the right upper corner are the percentage of the diseases that varies according to the approach. We clarified in L238-239. The size of each rectangle is relative to the percentage of the burden of each disease. |
